# Breaking Barriers: Investigating Gender Representation in the First Authors of Cardiovascular Disease and Artificial Intelligence Publications

**DOI:** 10.7759/cureus.45695

**Published:** 2023-09-21

**Authors:** Sampda Sharma, Anurag A Anghole, Snehal B Pathare, Manasi R Nagare, Shreya Choubey, Atiya Malik

**Affiliations:** 1 Internal Medicine, Gujarat Medical Education and Research Society (GMERS) Medical College, Gandhinagar, IND; 2 Internal Medicine, Smolensk State Medical University, Smolensk, RUS; 3 Internal Medicine, Smt Mathurabai Bhausaheb Thorat (SMBT) Institute of Medical Sciences and Research Centre, Nashik, IND; 4 Internal Medicine, AMA College of Medicine, Manila, PHL; 5 Internal Medicine, I.K. Akhunbaev Kyrgyz State Medical Academy, Bishkek, KGZ

**Keywords:** pubmed, ai and cardiovascular disease, research, gender equality, female

## Abstract

Introduction

Artificial intelligence (AI) and cardiovascular diseases have resulted in significant advancements in healthcare and medical research. This study focused on examining the gender equality ratio of first authors in "artificial intelligence and cardiovascular disease" articles from 2005 to 2022. It is critical to investigate gender representation in this dynamic subject given the growing usage of AI in cardiovascular medicine.

Aims

The aim of this study is to visualize the changing face of gender equality within the field of artificial intelligence (AI) and cardiovascular diseases by examining the gender distribution of the first authors' published articles from 2005 to 2022, providing important insights into disparities in gender and the potential for fostering inclusivity and diversity in the scientific community.

Methodology

All academic articles published from 2005 to 2022 were reviewed. The gender of the first author of each study was recorded. Since there were so few articles available for five months in 2023, they were excluded. The research was subsequently categorized based on the gender, ethnicity, and country of origin of the first authors.

Results

With a value of 0.54, the overall gender ratio favored male authors (275) over female authors (149). In 2022, female first authors had the most publications (59), while male first authors contributed 113 articles. Predictions for 2027 showed a significant increase in the number of publications on this topic by male authors (950) and female authors (580).

A gradual increase in the number of female first authors was observed over this period, although their representation remained lower compared to male first authors.

Conclusions

In the first authorship, our analysis found a gender gap, with male authors predominating. Females' engagement must be encouraged if academic gender equality is to be achieved. Female researchers are empowered by creating an inclusive atmosphere through mentorship and regulatory changes. For knowledge to advance fairly, collaboration is essential.

## Introduction

The intersection of artificial intelligence (AI) and cardiovascular diseases has yielded remarkable progress in medical research and patient care. With the expanding use of AI in cardiovascular medicine, it is crucial to investigate gender representation within this dynamic field [1]. Examining the gender ratio of first authorship in articles focused on AI applications in cardiovascular diseases from 2005 to 2022 can offer valuable insights into gender disparities and opportunities for fostering diversity and inclusivity in the scientific community.

Despite notable advancements in promoting females' inclusion in science and medicine, a persistent underrepresentation of females in academic publications persists. Authorship and opportunities are influenced by economic factors and geographic location. Examining the first authors' gender ratios is vital for understanding academic publishing gender disparities and barriers. Attempts to enhance females' representation in science, technology, engineering, and mathematics (STEM) fields have yielded some positive results. And it was concluded that between 1970 and 2004, the proportion of females serving as lead authors in first authorship increased significantly from 5.9% to 29.3% [2]. However, since 2004, this positive trend has stagnated, indicating a plateau in the representation of females as lead authors. The undervaluation of females' contributions to academic medicine and their lesser recognition on social media platforms can hinder their progress and career advancement [3]. It can also inform strategies to enhance gender equity, encourage females' participation, and create a more inclusive research environment [4]. Identifying and addressing barriers hindering the academic advancement of female physician-scientists are equally essential for progress in cardiology [5].

This study seeks to contribute to the wider conversation on gender equality in academia by examining the gender dynamics in AI and cardiovascular medicine research. With a specific focus on the significance of first authorship as an indicator of primary research responsibility, our investigation aims to shed light on potential gender disparities in authorship. Ultimately, by advancing gender representation in academic publications, scientific knowledge can be enhanced and positively impact patient outcomes.

The aim of this study is to evaluate the gender distribution of first authors in articles about cardiovascular disease and artificial intelligence that were published between 2005 and 2022 and to find any noteworthy trends or modifications in the gender distribution of initial authors throughout the given time frame.

## Materials and methods

This is a bibliometric analysis conducted on 27 June 2023. The PubMed database was searched for articles related to AI and cardiovascular diseases, using the Boolean operators "Artificial Intelligence" and "Cardiovascular disease." The date range was selected, and all articles published in the past 10 years from 1 January 2003 to 31 December 2022 were taken into consideration. Publications from the year 2023 were excluded, as only a few publications for five months were available. Articles accepted in the year 2022, which were published and appeared on PubMed in 2023, were included in the study. These were downloaded as comma-separated values (CSV) files. The total number of entries was equally divided among all six authors.

Each author scrutinized if the articles were relevant to the topic and noted the complete name of the first author via PubMed and the country. The Namsor application (Namsor, Paris, France) was used to find the gender of the first author [6]. The Namsor software correctly categorizes personal names based on gender, ethnicity, and country of origin. Statistical analysis was done using the R software (R Foundation for Statistical Computing, Vienna, Austria), autoregressive integrated moving average (ARIMA) model, and graphs prepared using Datawrapper (Datawrapper, Berlin, Germany).

## Results

**Figure 1:**
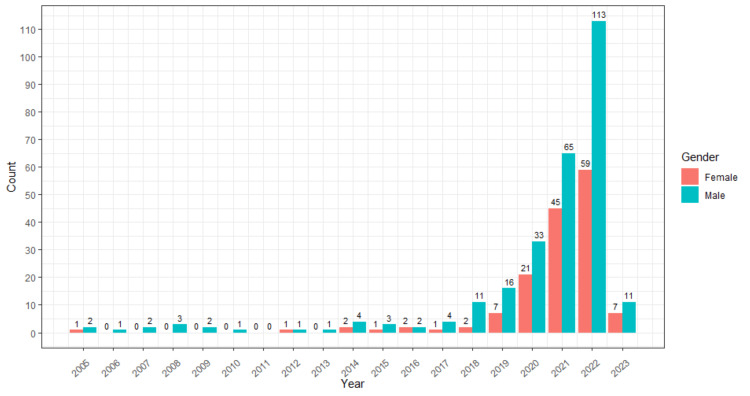
Total number of male and female first authors based on year.

Out of the total 425 first authors, there were 275 males and 149 females. Figure 1 shows the total number of male and female first authors based on year. In artificial intelligence and cardiovascular disease, the year 2022 saw the highest number of female first authors with 59 articles.

**Figure 2:**
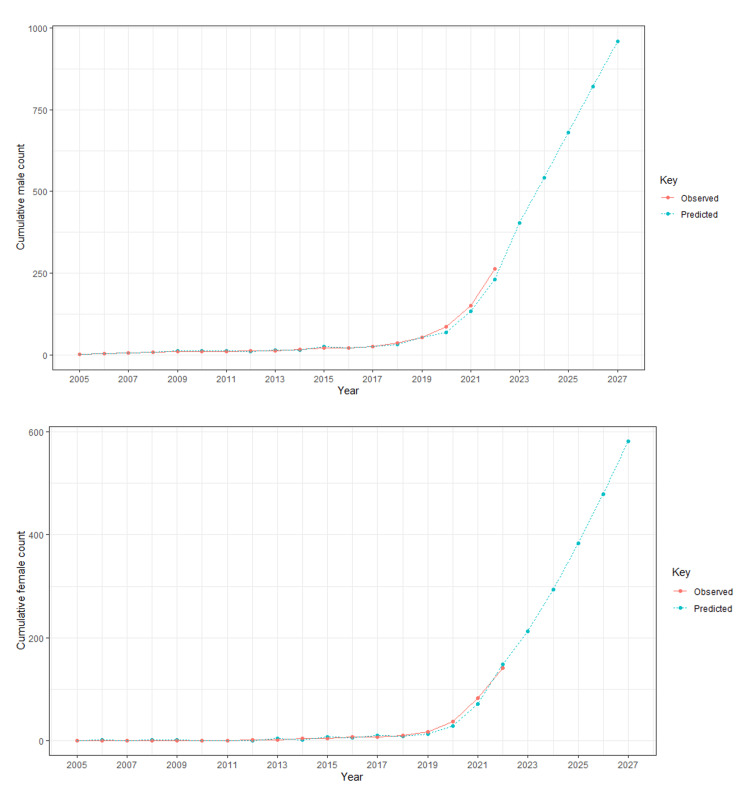
Cumulative male and female count forecasting.

Figure 2 shows the observed publication trends among cumulative male count (upper image) and cumulative female count (lower image) of first authors from 2005 to 2022 and the prediction of trends for the next five years. Around 900 publications are anticipated to be written by male first authors in 2027, compared to around 600 publications written by females. The ARIMA model was used to calculate this, and modelling data from the years 2005 to 2022 were used. From the year 2005 to the year 2027, forecasting was done.

**Figure 3:**
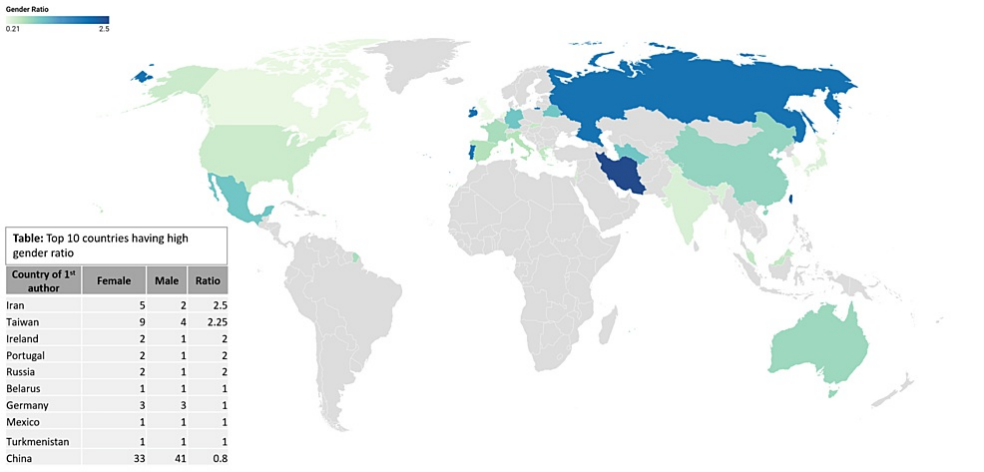
Gender trends in publications (2005-2023) based on the country.

Figure 3 shows the gender trends in publications (2005-2023) based on country. It demonstrates that Iran has the highest gender ratio of 2.5, followed by Taiwan, which is 2.25. This chart was prepared using Datawrapper.

Fisher's exact test is performed for the gender and country variables. A p value of 0.1354 was obtained, which signifies that there is no significant association present between gender and country.

Table 1 shows the top journals having favorable gender ratios. It demonstrates that the European Heart Journal Digital Health has the most favorable gender ratio.

**Table 1:** Top journals having favorable gender ratio.

Journal	Female	Male	Ratio
Eur Heart J Digit Health	3	3	1
Am J Obstet Gynecol	1	1	1
BMC Cardiovasc Disord	1	1	1
BMC Health Serv Res	1	1	1
BMC Med Inform Decis Mak	1	1	1
Cells	1	1	1
Circ Res	1	1	1
Clin Kidney J	1	1	1
Ebiomedicine	1	1	1
Front Oncol	1	1	1

Table 2 shows the top countries having high gender ratio (at least 10 publications). It demonstrates that Taiwan has a high gender ratio of 2.25, followed by China, which has a ratio of 0.8.

**Table 2:** Top countries having a high gender ratio (at least 10 publications).

Country of the first author	Female	Male	Ratio
Taiwan	9	4	2.25
China	33	41	0.8
Spain	6	10	0.6
Italy	7	12	0.58
United States of America	30	67	0.45
India	5	14	0.36
Japan	3	9	0.33
Netherland	3	11	0.27
South Korea	2	8	0.25
United Kingdom	6	28	0.21

Table 3 shows the top journals having a high gender ratio. It demonstrates that the International Journal of Molecular Sciences has the highest ratio of 4, followed by the International Journal of Environmental Research and Public Health, which has a ratio of 2.33.

**Table 3:** Top journals having a high gender ratio.

Journal	Female	Male	Ratio
Int J Mol Sci	4	1	4
Int J Environ Res Public Health	7	3	2.33
Am Heart J Plus	2	1	2
Ann Transl Med	2	1	2
Biomedicines	2	1	2
Eur Radiol	2	1	2
J Med Internet Res	2	1	2
JMIR Med Inform	2	1	2
Nutrients	2	1	2
J Clin Med	3	2	1.5

## Discussion

This study explored the gender ratio of first authors in "artificial intelligence and cardiovascular disease" articles from 2005 to 2022. The overall gender ratio favored male authors (275) over female authors (149), with a value of 0.54. The highest number of publications for the female first author was in the year 2022, and the number is 59, while male first authors contributed 113 articles. Predictions for 2027 suggested a substantial increase in publications by male authors (950) and female authors (580) on this topic. Iran had the highest gender ratio (2.5 females to one male) for first authors, followed closely by Taiwan (2.25). In contrast, China exhibited the lowest gender ratio (0.8 females to one male) in academic publications (Figure 3). When considering studies with at least 10 publications, Taiwan emerged as the country with the highest gender ratio (2.25), followed by China (0.8), while the United Kingdom had the lowest ratio (0.21) (Table 2). The study identified certain journals that demonstrated balanced gender representation (Table 1), while others showed a high gender ratio (Table 3). Through Fisher's exact test analysis, no substantial association was found between the gender of the first authors and their countries (p value = 0.1354). This suggests that the distribution of gender in the first authorship was not significantly influenced by the authors' countries of residence.

Comparison with other studies

This study focused on examining the gender equality ratio of first authors in "artificial intelligence and cardiovascular disease" articles from 2005 to 2022. A gradual increase in the number of female first authors was observed over this period, although their representation remained lower compared to male first authors. These findings align with previous research by Brown et al. (2020), who highlighted gender disparity in orthopedic literature from 1987 to 2017 [7]. Similarly, Polanco et al. (2020) conducted a study on gender trends in hepatology publications, revealing a significant difference with 65.1% male first authors and 34.9% female first authors [8].

In this study, a noteworthy increase was observed in the number of female first authors over the years, rising from one in 2005 to 59 in 2022. This trend aligns with the findings of Filardo et al. (2016), who reported a significant rise in female first authorship in high-impact medical journals from 1994 (27%) to 2014 (37%) [9].

Similarly, Phurtag et al. (2022) conducted a study on gender equality trends in spine research publications and noted a significant increase in female first authorship from 0% to 19.8% between 1976 and 2020 [10]. However, while progress has been observed in first authorship, this study and that of Phurtag et al. [10] found that the rise in senior authorship for females was not statistically significant (from 4.3% to 14.4%). This highlights the need for targeted efforts to bridge the gender gap in senior authorship positions and foster a more equitable representation of females across all levels of academic research.

Several studies have explored gender equality trends in authorship across various medical fields. Okike et al. (2012) investigated orthopedic journals and found that the representation of females increased from 1970 to 2007. The first authors rose from 0.8% to 6.5%, last authors from 0.0% to 4.3%, and editorial board members from 1.6% to 5.4%. However, orthopedics' rate of increase was lower than other medical fields [11].

Similarly, Fishman et al. (2017) analyzed pediatric journals, revealing an increasing trend in female representation for first authors (from 39.8% to 57.7%), senior authors (from 28.6% to 38.1%), and editorial boards (from 17.8% to 39.8%) over time [12].

Jagsi et al. (2006) focused on academic medical literature and observed a rise in female first authors from 5.9% to 29.3% and female senior authors from 3.7% to 19.3% between 1970 and 2004 [13].

Conversely, O'Connor et al. (2018) examined radiology publications in the United States of America from 1970 to 2016, noting that while female radiologists had steady increases in authorship until 2000, there was a subsequent decrease in female first and corresponding authorships (from 0.81 to 0.34), resulting in a 47% drop in the number of female first authors added per year [14].

In this study, a forecast was conducted for the number of male and female first authors in the next five years, revealing a significant increase. Male first authors are projected to rise from 275 (in 2022) to around 950 (in 2027), while female first authors are expected to increase from 149 (in 2022) to around 580 (in 2027).

A similar study by Rickard et al. (2020) examined trends in pediatric urology and predicted that by the year 2049, there will be 55% female corresponding authors and 83% female first authors [15].

Regarding the gender ratio of first authors by country, this study identified Taiwan (2.25) with the highest female to male ratio, followed by China (0.8). In contrast, South Korea (0.25) and the United Kingdom (0.21) had the lowest ratios. At least 10 publications were considered for this analysis.

Phurtag et al. conducted a study on the country-wise distribution of female authors in spine research publications, showing contrasting results. The Netherlands and Canada exhibited higher percentages of female authors, with 33.8% and 28.2%, respectively. Meanwhile, Japan and Korea showed the lowest number of female authors, with 7.1% and 3.2%, respectively [10].

Limitations

The limitations of this study are the following: reliance on PubMed data, which may have introduced potential gaps or inconsistencies; focus on first authors, potentially not fully representing gender distribution across all author positions; limited scope, covering specific journals and a particular time frame, possibly not capturing the entire gender landscape in academic publications; uncertainty in gender identification due to the use of the Namsor application, which may introduce some level of uncertainty; and missing gender information for certain authors, potentially influencing the overall results.

## Conclusions

This study revealed a gender disparity in first authorship, with male authors dominating. Encouraging females' participation is vital for academic gender equality. Creating an inclusive environment through mentorship and policy changes empowers female researchers. Collaborative efforts are crucial for advancing knowledge equitably.
